# Supplementary material for: Reversed size-dependent stabilization of ordered nanophases
Source: Nat Commun. 2019 Apr 30;10:1982. doi: 10.1038/s41467-019-09841-3 (PMC6491558; doi:10.1038/s41467-019-09841-3)
Supplement: Supplementary file 1 — Supplementary Information [file 41467_2019_9841_MOESM1_ESM.pdf]

Reversed size-dependent stabilization of ordered nanophases

Pirart et al.

Supplementary Note 1: Experiments

In Supplementary Figure 1 we report TEM images and data about as-grown room-temperature samples and 400 ^{o}C annealed samples.

In Supplementary Figure 2 we report the results of GISAXS measurements, which were performed on the 3.0 10^{15} at.cm^{-2} AgPt sample. This technique [1,2] allows to probe a high number of nanoparticles ($\sim 10^9$ NPs) to determine their 3D morphological features (shape, size and interparticle distance). From 2D GISAXS patterns (Supplementary Figure 2(a,b)), two intensity cuts, i.e. two cross sections in the q_z and q_y directions were extracted. These cuts are simultaneously fitted with the IsGISAXS software [3]. GISAXS measurements were performed in situ after growth at room temperature (Supplementary Figure 2(a,c,d)) and after annealing up to 400 ^{o}C (Supplementary Figure 2(b,c,d)).

Supplementary Table 1: GISAXS data for the sample with flux 3.0 10^{15} at.cm^{-2} and composition 55% at.Ag. D and H are the average diameter and height of the nanoparticles, respectively. σ_D is the standard deviation of the diameter.

	D (nm)	H/D	σ_D/D
Room temperature	1.64±0.05	0.76±0.03	0.30±0.02
400 ^{o}C	2.44±0.05	0.82±0.03	0.32±0.02

Supplementary Note 2: DFT calculations

In order to check the possible role of twinning in stabilizing cluster structures, we consider $Ag_{67}Pt_{12}$, comparing the $L1_1$@Ag skin structures without and with a twin plane (the latter denoted as $L1_1^t$@Ag structure). Both structures are shown in Supplementary Figure 3. $L1_1^t$@Ag is obtained from perfect $L1_1$@Ag by two atomic pair swaps, and it is higher in energy by 0.04 eV only. Note that a single atomic swap to bring a Pt atom on the surface would cost 0.24 eV at least. This

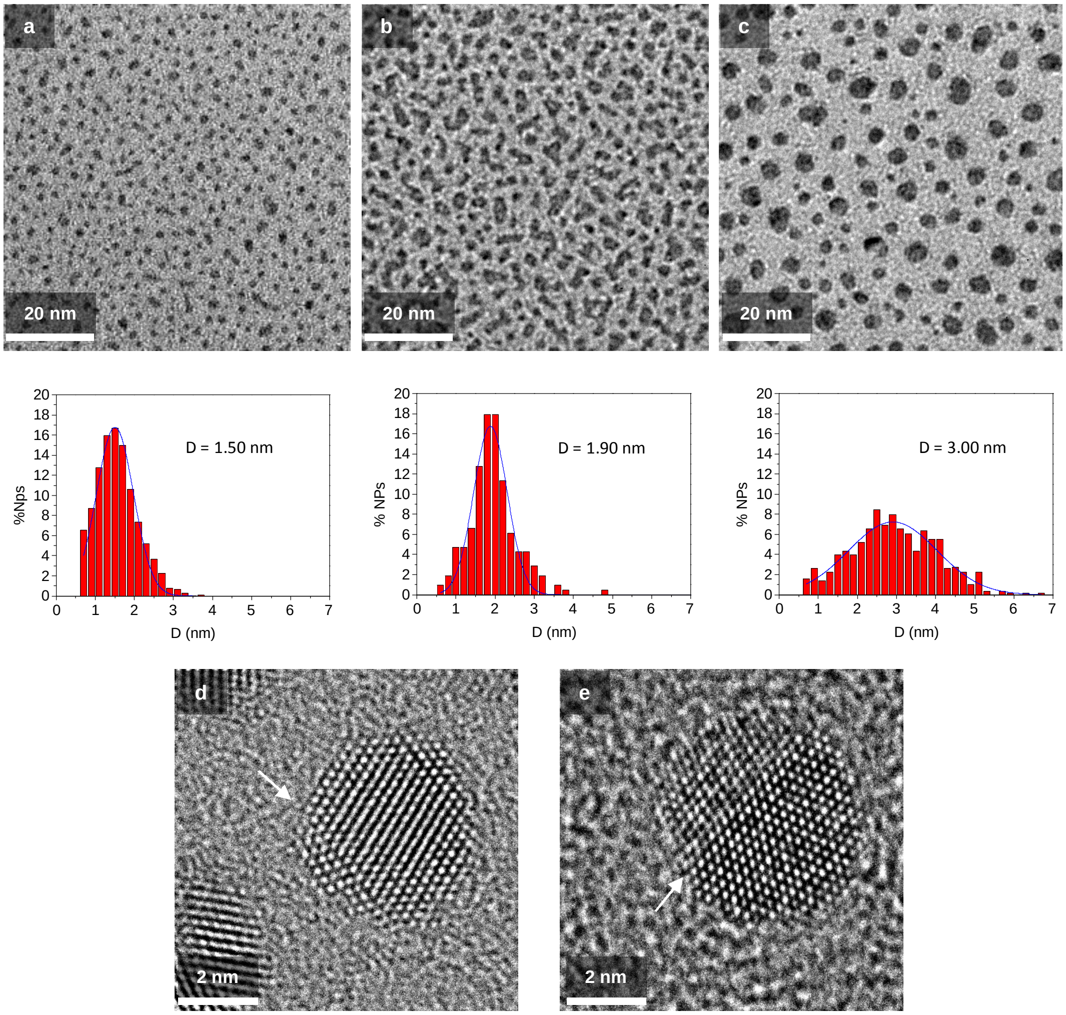

Supplementary Figure 1: TEM images and their corresponding size histograms of AgPt samples with (a) 2.3 10^{15} at.cm^{-2}, (b) 3.2 10^{15} at.cm^{-2} at room temperature and (c) 3.2 10^{15} at.cm^{-2} after annealing at 400 ^{o}C. (d) and (e) HRTEM images after annealing at 400 ^{o}C of the 3.2 10^{15} at.cm^{-2} AgPt sample showing single particles with twin boundaries.

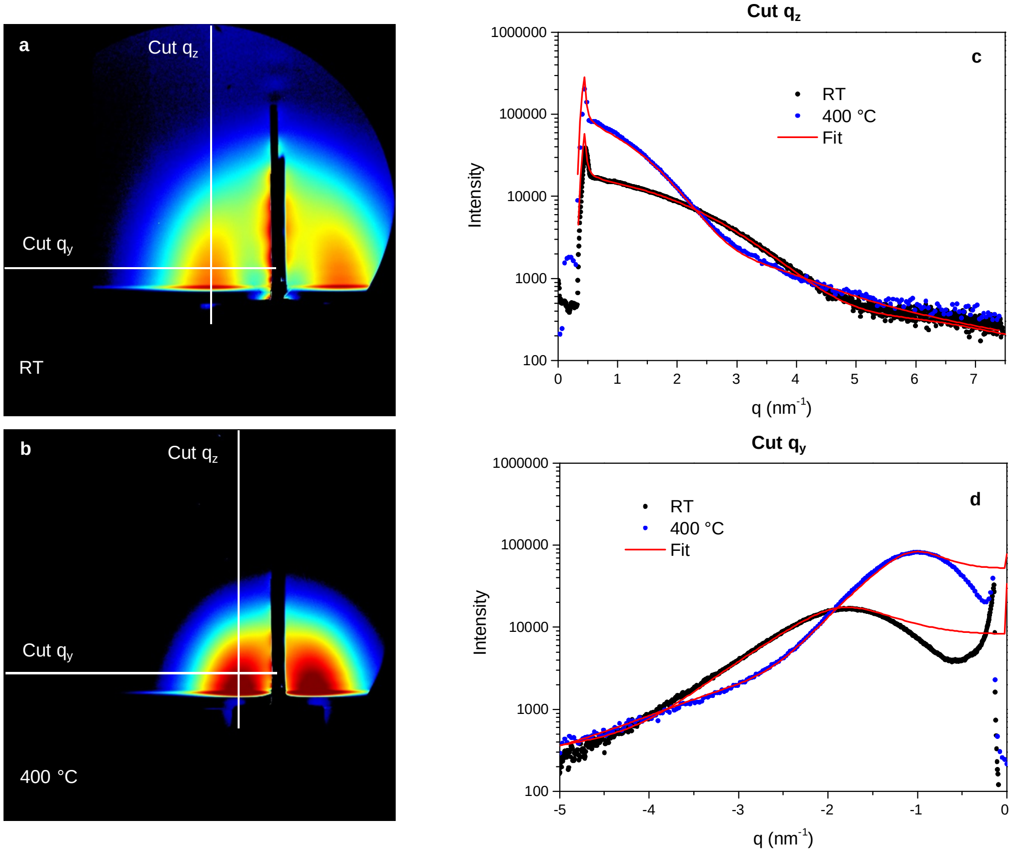

Supplementary Figure 2: GISAXS patterns of AgPt sample with 3.0 10^{15} at.cm^{-2} (a) at room temperature and (b) at 400 ^{o}C. The white lines represent the position of the q_z and q_y cuts that are respectively shown in (c) and (d) with their corresponding fits. The parameters used in the simulation of the fitted curves are listed in the table below.

indicates that energy differences related to surface segregation are much larger than those related to twinning. This point is supported also by our atomistic model, and by the DFT results shown in the following.

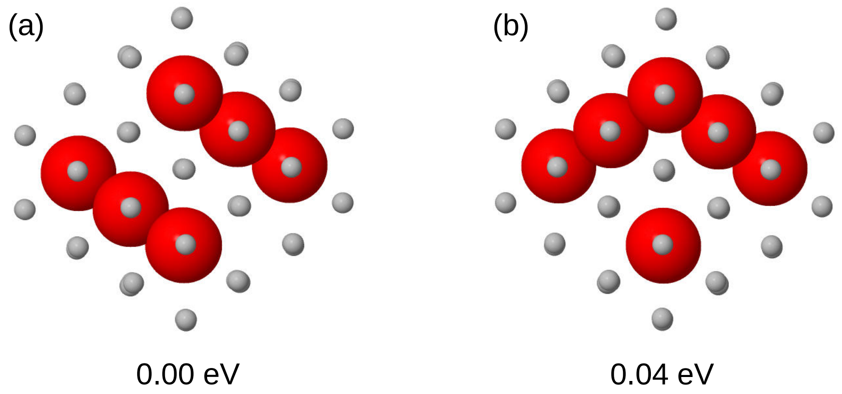

Supplementary Figure 3: (a) $L1_1$@Ag structure and (b) $L1_1^t$@Ag structure.

In Supplementary Figure 4 we consider the energetic stability of the perfect $L1_1$ phase without Ag skin, which, in the 79-atom truncated octahedron, can be obtained for composition $Ag_{43}Pt_{36}$ (with Ag/Pt/Ag/Pt/Ag planes, Supplementary Figure 4(a)) and composition $Ag_{36}Pt_{43}$ (with Pt/Ag/Pt/Ag/Pt planes, Supplementary Figure 4(c)). For both cases, we made global optimization runs of chemical ordering by 10^3 unlike atom pair swaps, which produced finally structures (b) and (d). Structure (b) is lower in energy than (a) by 2.00 eV, whereas structure (d) is lower in energy than structure (c) by 5.99 eV. These results clearly show that the $L1_1$ phase without Ag skin is strongly disfavoured from the energetic point of view.

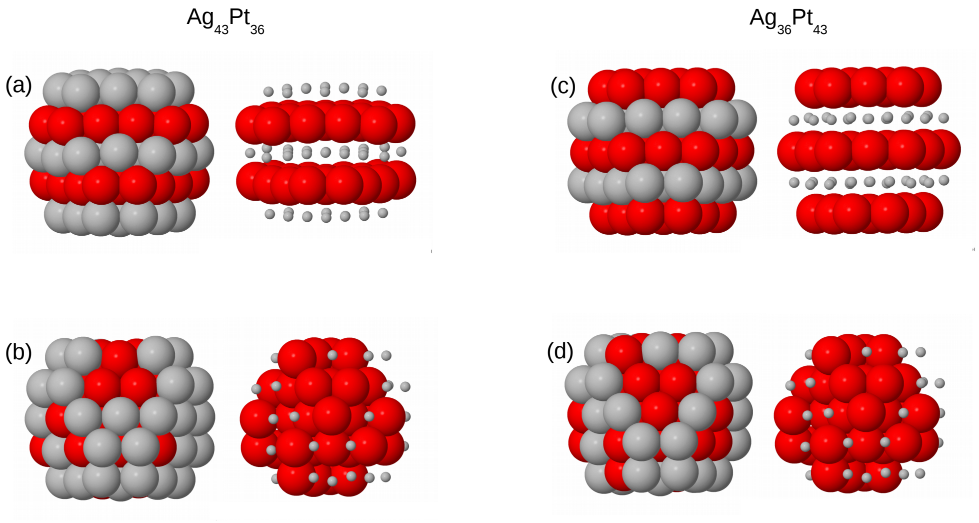

Supplementary Figure 4: DFT chemical ordering optimization results for truncated octahedral $Ag_{43}Pt_{36}$ and $Ag_{36}Pt_{43}$. Pt atoms are in red and Ag atoms in grey. Each cluster is shown in two views. In the second view, Ag atoms are shown small spheres. (a) Perfect $L1_1$ phase with Ag/Pt/Ag/Pt/Ag alternating (111) planes at composition $Ag_{43}Pt_{36}$. (b) Best structure found for $Ag_{43}Pt_{36}$. (c) Perfect $L1_1$ phase with Pt/Ag/Pt/Ag/Pt alternating (111) planes at composition $Ag_{36}Pt_{43}$. (d) Best structure found for $Ag_{36}Pt_{43}$.

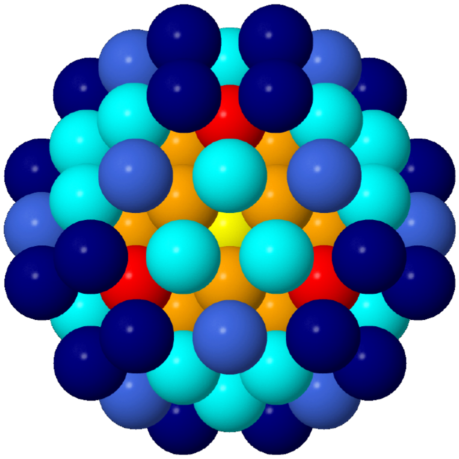

same color, equivalent position

	Pt position	(QE) ΔE[eV]	(CP2K) ΔE[eV]
(orange)	*Inside* Edge	0	0
(red)	*Inside* Vertex	0.060	0.068
(cyan)	*Surface* Tri	0.284	0.336
(yellow)	*Inside* Central	0.356	0.305
(blue)	*Surface* Edge	0.365	0.393
(dark blue)	*Surface* Vertex	0.610	0.691

ΔE respect to the best energy

Supplementary Figure 5: Energies of the inequivalent placements of the a single Pt impurity in a truncated octahedron of 79 atoms (composition $Ag_{78}Pt_1$). There are six inequivalent positions for the impurity, three of them are inside the cluster (one central position and two subsurface positions), the remaining three are on the surface. The two subsurface positions are the lowest in energy. Both Quantum Espresso (QE) and CP2K results are given.

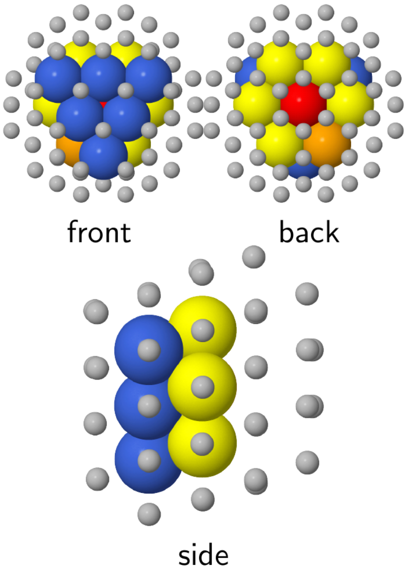

	Pt position	(QE) ΔE[eV]	(CP2K) ΔE[eV]
	Lateral	0	0
	Central	0.599	0.624
	Central "Donut"	0.625	0.745

ΔE respect to the best energy

Supplementary Figure 6: Energies of the inequivalent positions to place a leaflet of six Pt atoms in a truncated octahedron of 79 atoms (composition $Ag_{73}Pt_6$). There are three inequivalent positions for the leaflet, one of them is subsurface and two of them are central. The subsurface position is the lowest in energy. Both Quantum Espresso (QE) and CP2K results are given.

In Supplementary Figure 5 we report the DFT energies of all different inequivalent placements of a single impurity in a truncated ochahedron of 79 atoms (composition $Ag_{78}Pt_1$), and in Supplementary Figure 6 and of the inequivalent configurations of a Pt leaflet of six atoms againin a truncated ochahedron of 79 atoms (composition $Ag_{73}Pt_6$). In both cases, subsurface placements are more favourable, according to both Quantum Espresso and CP2K calculations.

Supplementary Note 3: Pt-Ag interatomic potential

The semi-empirical many-body potential used to resolve the chemical ordering of large-size nanoparticles is the one derived from the tight binding model in which the density of states is approximated to its second moment (Second Moment Approximation "SMA") [4] so that the attractive term comes from the d-band of the metal and the many-body character appears with the square root dependence of the number of bonds. A repulsive empirical term is added to take into account the core electrons repulsion. For the Pt-Ag system, we have added a third term (a gaussian) to the SMA potential, in order to stabilize the $L1_1$ phase, at the equiconcentration, instead of the usual $L1_0$ ordered phase. The $L1_1$ phase is characterized by an alternance of pure atomic planes in the (111) direction whereas the $L1_0$ phase displays this alternance in the (100) direction. The stabilization of the $L1_1$ phase is due to a reinforcement of the second-neighbour mixed interactions as compared to the first-neighbours ones [5]. The semi-empirical potential is thus expressed as follows:

$$E_i^a = -\sqrt{\sum_{j, r_{ij} < r_c} \xi_{ab}^2 \mathrm{e}^{-2q_{ab}(\frac{r_{ij}}{r_1}-1)}} + \sum_{j, r_{ij} < r_c} A_{ab} \mathrm{e}^{-p_{ab}(\frac{r_{ij}}{r_1}-1)} - \sum_{j, r_{ij} < r_c} \Gamma \mathrm{e}^{-\frac{(r_{ij}-r_2)^2}{2\sigma^2}} \quad (1)$$

where r_{ij} is the distance between the atom at site i and their neighbors at site j, r_c is the cut-off distance and r_1, r_2 are the first and second neighbor distance, and p_{ab}, q_{ab}, A_{ab}, ξ_{ab}, Γ and σ are six parameters (listed in Table 2). The parameters for the pure metals have been fitted to the DFT-PBE values of the cohesive energies and lattice parameters of Pt and Ag and to the experimental values of their elastic constants. The parameters for the Pt-Ag interactions have been fitted to reproduce at the best the mixing enthalpy of the $L1_1$ phase obtained from DFT-PBE calculations (Table 3). The mixing enthalpy is the energy difference between the chemically ordered phase ($L1_1$ or $L1_0$)

and the demixed alloy (i.e. the sum of the cohesive energy of the pure metals). We notice on the Table 3 that the mixing enthalpy of the $L1_0$ phase is positive and the SMA potential reproduces quite well the DFT-PBE result, whereas the mixing enthalpy of the $L1_1$ phase is negative but the SMA potential overestimates it by a factor of four. This overestimate is necessary to fit in the same time the experimental order/disorder critical temperature which is equal to 1200 K. Such quantity is important to also model the effect of the temperature on the clusters ordering.

Supplementary Table 2: Parameters of the Pt-Ag modified SMA potential

$a-b$	p_{ab}	q_{ab}	A_{ab}	ξ_{ab}	Γ	σ	a_{ab}	E_{ab}^{coh}
Pt-Pt	10.7960	3.1976	0.199272	2.231779	-	-	3.98	-5.53
Ag-Ag	11.7240	2.8040	0.074780	1.006360	-	-	4.16	-2.725
Pt-Ag	11.2600	3.0008	0.145600	1.592000	0.044	0.035	-	-

Supplementary Table 3: Mixing enthalpies of PtAg alloys.

ΔH_{mixing} (meV/at.)	$L1_1$	$L1_0$
DFT-PBE	-51	41
SMA	-213	63

Supplementary References

1. Lazzari, R., Leroy, F. & Renaud, G. Grazing-incidence small-angle x-ray scattering from dense packing of islands on surfaces: Development of distorted wave born approximation and correlation between particle sizes and spacing. *Phys. Rev. B* **76**, 125411 (2007).

2. Andreazza, P. Probing nanoalloy structure and morphology by x-ray scattering methods (2012). In *Nanoalloys – Synthesis, Structure and Properties*, pages 69-112 . Edited by D. Alloyeau, C. Mottet and C. Ricolleau. Springer Verlag, Berlin.

3. Lazzari, R. *IsGISAXS*: a program for grazing-incidence small-angle X-ray scattering analysis of supported islands. *Journal of Applied Crystallography* **35**, 406–421 (2002).

4. Rosato, V., Guillopé, M. & Legrand, B. Thermodynamical and structural properties of f.c.c. transition metals using a simple tight-binding model. *Phil. Mag. A* **59**, 321–336 (1989).

5. Khoutami, A., Legrand, B. & Tréglia, G. On a surprising anisotropy of surface segregation in cupt alloys. *Surf. Sci.* **287/288**, 851–856 (1993).
